# Sub genomic analysis of SARS-CoV-2 using short read amplicon-based sequencing

**DOI:** 10.3389/fgene.2023.1086865

**Published:** 2023-02-24

**Authors:** Lian Chye Winston Koh, Yiqi Seow, Kiat Whye Kong, Ming Li Lalita Lau, Shoban Krishna Kumar, Gabriel Yan, Chun Kiat Lee, Benedict Yan, Paul Anantharajah Tambyah, Shawn Hoon

**Affiliations:** ^1^ Bioinformatics Institute, Agency for Science Technology and Research, Singapore; ^2^ Institute for Bioengineering and Bioimaging, Agency for Science Technology and Research, Singapore; ^3^ Institute for Molecular and Cell Biology, Agency for Science Technology and Research, Singapore; ^4^ Division of Advanced Internal Medicine, Department of Medicine, National University Health System, Singapore; ^5^ Division of Infectious Diseases, Department of Medicine, National University Health System, Singapore; ^6^ Molecular Diagnosis Centre, Department of Laboratory Medicine, National University Health System, Singapore

**Keywords:** genomics, SARS-CoV-2, subgenomic RNA, transcriptome, temporal

## Abstract

The novel coronavirus disease 2019 (COVID-19) pandemic poses a serious public health risk. In this report, we present a modified sequencing workflow using short tiling (280bp) amplicons library preparation method paired with Illumina’s iSeq100 desktop sequencer. We demonstrated the utility of our workflow in identifying gapped reads that capture characteristics of subgenomic RNA junctions within our patient cohort. These analytical and library preparation approaches allow a versatile, small footprint and decentralized deployment that can facilitate comprehensive genetics characterizations during outbreaks. Based on the sequencing data, Taqman assays were designed to accurately capture the quantity of subgenomic ORF5 and ORF7a RNA from patient samples and demonstrated utility in tracking subgenomic titres in patient samples when combined with a standard COVID-19 qRT-PCR assay.

## 1 Introduction

SARS-CoV-2 patients present with a range of severity and it is unclear how infectious asymptomatic cases are although they are believed to contribute to the spread of the virus. The proportion of asymptomatic patients have been estimated to range from 40%–45% and tend to be younger and do not have preexisting conditions (Oran and Topol, 2020). Conversely, there are many patients who remain PCR positive for weeks despite clinically recovering from the infection. This has posed an important question from a public health management perspective: When can we discharge or remove from isolation patients who are clinically well but remain PCR positive? Increasingly, the consensus is that PCR tests are picking up viral debris shed after an infection and that a time-based discharge criterion may be a more appropriate measure. WHO currently recommends that patients who are clinically well can be discharged 10 days after their symptoms began as long as they have been symptom-free for three consecutive days. Most countries now release patients from isolation between 7 and 14 days from onset of symptoms (He et al., 2020; MIN COW, 2020). In Singapore, COVID-19 patients who are clinically well are discharged at day 21 of their illness without the need for repeat PCR testing, though immunocompromised patients still require two negative PCR tests before discharge (Ministry of Health, 2022).

Coronaviruses express 3’ proximal ORFs through subgenomic RNAs (sgRNAs) that are generated by a discontinuous transcription mechanism that is unique to RNA viruses (Sola et al., 2015). A study from Germany showed that infectious virus was isolated from throat or lung but not from stool samples and this correlated with evidence of active replication in throat samples by measuring the levels of transcribed subgenomic mRNA which are only produced by infected cells (Wölfel et al., 2020). The present study examines the expression of subgenomic RNAs in 53 patients. In addition, we demonstrated the ability of our workflow to capture temporal dynamics of amplicon coverage and subgenomic RNA junctional reads during an infection time course over 15 days.

## 2 Materials and methods

### 2.1 Patient samples

53 patients admitted to the National University Health System, from Feb to May 2020, with SARS-CoV-2 confirmed by RT-PCR (MiRXES, Singapore [Feb-Mar]; Cobas SARS-CoV-2 Roche, Switzerland [from Apr onwards]) from nasopharyngeal swabs were included in this study. Ethics approval was obtained from the National Healthcare Group Institutional Review Board.

### 2.2 Reverse transcription and quantitative PCR

400 µl of nasopharyngeal swab solubilized in UTM was extracted using EZ1 virus mini kit (Qiagen) into 90 µl elution buffer. An aliquot was heat-inactivated at 60°C for 30 min as required by Ministry of Health (Singapore) to prevent SARS-COV-2 infection and 6.5 µl of the heat-inactivated nucleic acid was reverse transcribed using SuperScript III (Life Technologies) (2 µl 5x FS buffer, 0.5 µl dNTPs, 0.5 µl 0.1M DTT, 0.25 µl SuperScript III) using 0.25 µl of Random Primer mix (NEB) using the following protocol: 65°C 20s, 4°C 60s, 55°C 50 min, 95°C 5 min.

### 2.3 Pooled PCR and library preparation

1 µl of the cDNA was then used for each pooled PCR in the following 10 µl Q5 polymerase reaction mix (NEB) (1 µl 10x Q5 buffer, 0.8 µl 10 mM dNTPs, 0.5 µl 10 µM pooled primers (Pool 1 or 2), 0.1 µl Q5 polymerase) using cycling protocols of 98°C 15s, 63°C 3 min s, 38 cycles). The two pools were combined after PCR and purified using 18 µl AmpureXP beads (Beckman-Coulter) as per manufacturer’s protocol and eluted in 20 µl water. 10ng of the amplified product was prepared for library with ligation and barcoded using the NEXTFLEX Rapid DNA Seq 2.0 kit (Perkins-Elmer) as per manufacturer’s instructions and eluted in 25 µl of water. Samples were pooled at 4-6 samples per run and diluted to 50pM before loading into the iSeq100 cartridge (150 nt read, paired end; Illumina) as per manufacturer’s instructions.

### 2.4 Read processing, alignment and quality control

The critical step in the analysis is the alignment of partial transcript reads spanning sub genomic junctions to the reference genome. To achieve this, we developed a gapped alignment workflow that combines GSNAP (Wu and Watanabe, 2005) and V-ASAP (Maurier et al., 2019) to identify and quantify reads spanning these sub-genomic junctions. GSNAP was deployed over other available tools such as TOPHAT because it allows for an unbiased approach by not requiring an existing annotation of the subgenomic transcripts. Following alignment with GSNAP, gapped reads were filtered from the resulting SAM files based on CIGAR (Compact Idiosyncratic Gapped Alignment Report) strings using an AWK script. The remaining non-gapped reads are processed using V-ASAP, which merges the overlapping paired end reads and quality controls the merged amplicon reads to specifically those containing our intended forward and reverse primer sequences. Merged amplicon reads that passed quality control is realigned to the genome using BWA (Li and Durbin, 2009), this is followed by variant calling using SAMTOOLS and BCFTOOLS (Li et al., 2009).

The filtered gapped read SAM entries from GSNAP alignment, were further narrowed down to those spanning the known canonical subgenomic regions using RSAMTOOLS in R (Bioconductor, 2022). The quantity of these reads is then normalized by sequencing depth for comparison across the sample cohort and time points. Visualization of the downstream characterization are plotted using GGPLOT2 (Wickham, 2009). Reads Counts for subgenomic reads are represented in terms of reads per million of total sequenced reads to allow for easy reference to proportion of reads and also to account for the impact of sequencing depth.

### 2.5 Real-time PCR

Real-time PCR with SyBr Green was performed with 10 µl reactions using the FirePol MasterMix (Solis Biodyne) with 1 µl of 1:5 dilution of cDNA prepared previously for sequencing and 0.5 µl of 10 µM primers. For subgenomic RNA, we used covid_1 primer_left and covid_113/133/137/139/143/145/146/148 primer_right from the pool PCR primer set (Table 1) for qPCR.

**TABLE 1:** ARTIC primer design.

Primer name	Primer left	Primer right
covid_1	ACC​AAC​CAA​CTT​TCG​ATC​TCT​TGT	CTC​GTT​GAA​ACC​AGG​GAC​AAG​G
covid_2	CAG​CAC​ATC​TAG​GTT​TCG​TCC​G	CGA​GCA​TCC​GAA​CGT​TTG​ATG​A
covid_3	GGC​ACT​TGT​GGC​TTA​GTA​GAA​GT	AAA​TGA​CTT​TAG​ATC​GGC​GCC​G
covid_4	GGC​GAA​ATA​CCA​GTG​GCT​TAC​C	ATG​CAC​TCA​AGA​GGG​TAG​CCA​T
covid_5	TTA​ACG​GAG​GGG​CAT​ACA​CTC​G	GGA​CAT​TCC​CCA​TTG​AAG​GTG​T
covid_6	AGA​GCT​ATG​AAT​TGC​AGA​CAC​CTT	CGT​CTG​CCA​TGA​AGT​TTC​ACC​A
covid_7	GCG​TCA​CCA​AAT​GAA​TGC​AAC​C	ATA​GTG​CGA​CCA​CCC​TTA​CGA​A
covid_8	ACC​TGA​GCA​TAG​TCT​TGC​CGA​A	TGG​CGA​TCT​CTT​CAT​TAA​GTT​TAA​AGT​CA
covid_9	TGA​CAA​CCT​TCT​TGA​AAT​ACT​CCA​AAA​A	TAC​AAC​ACG​AGC​AGC​CTC​TGA​T
covid_10	GGT​GCC​TGG​AAT​ATT​GGT​GAA​CA	ACA​CCA​CCT​GTA​ATG​TAG​GCC​A
covid_11	ACT​GAG​ACT​CAT​TGA​TGC​TAT​GAT​GT	ACA​GGT​GAC​AAT​TTG​TCC​ACC​G
covid_12	AGA​GTT​TCT​TAG​AGA​CGG​TTG​GGA	GCA​TGA​GTA​GGC​CAG​TTT​CTT​CT
covid_13	ATT​TGT​CAC​GCA​CTC​AAA​GGG​A	TTT​CGA​GCA​ACA​TAA​GCC​CGT​T
covid_14	ACA​ACC​TAC​TAG​TGA​AGC​TGT​TGA​A	ACC​GAG​TTC​AAC​TGT​ATA​GGC​AGA
covid_15	TGA​ATA​TCA​CTT​TTG​AAC​TTG​ATG​AAA​GGA	ACC​TTC​TTC​TTC​ATC​CTC​ATC​TGG​A
covid_16	TGA​GTC​TGG​TGA​GTT​TAA​ATT​GGC​T	GAT​TGT​CCT​CAC​TGC​CGT​CTT​G
covid_17	TCA​ACC​TGA​AGA​AGA​GCA​AGA​AGA​A	TGG​CTG​CAT​TAA​CAA​CCA​CTG​T
covid_18	TGG​AAC​TTA​CAC​CAG​TTG​TTC​AGA​C	TGT​TTA​GCA​AGA​TTG​TGT​CCG​CT
covid_19	TGA​TGA​TTA​CAT​AGC​TAC​TAA​TGG​ACC​AC	ACA​GCT​AAG​TAG​ACA​TTT​GTG​CGA
covid_20	AGC​TGG​TAT​TTT​TGG​TGC​TGA​CC	GTG​AGG​AAC​TTA​GTT​TCT​TCC​AGA​GT
covid_21	CCT​TCA​GTT​GAA​CAG​AGA​AAA​CAA​GAT	CAG​TAG​TGC​CAC​CAG​CCT​TTT​T
covid_22	GGG​TGA​TGT​TGT​TCA​AGA​GGG​T	CAT​GTG​CAA​GCA​TTT​CTC​GCA​A
covid_23	AAA​AGT​GCC​TTT​TAC​ATT​CTA​CCA​TCT​ATT	GTG​TGT​TGA​TAA​GTG​ACG​CTA​CAG​T
covid_24	GGG​TGT​GGT​TGA​TTA​TGG​TGC​T	ACC​AGC​AAG​TGA​GAT​GGT​TTC​A
covid_25	ACA​GCG​TAT​AAT​GGT​TAT​CTT​ACT​TCT​TCT	CAC​AAC​TTG​CGT​GTG​GAG​GTT​A
covid_26	CTT​CTT​TCT​TTG​AGA​GAA​GTG​AGG​ACT	TGC​TGA​CAT​GTA​CCT​ACC​CAG​A
covid_27	CAC​TCT​ACG​TGT​TGA​GGC​TTT​TGA	GCA​CAA​AAG​TTA​GCA​GCT​TCA​CC
covid_28	AGT​TGA​AGT​TTA​ATC​CAC​CTG​CTC​T	GTG​TGC​CCA​TGT​ACA​TAA​CAG​CT
covid_29	ACG​TGG​TGT​GTA​AAA​CTT​GTG​GA	ACC​GTC​TAT​GCA​ATA​CAA​AGT​TTC​TTT
covid_30	TGG​TAC​ATT​TAC​TTG​TGC​TAG​TGA​GT	ACA​AGA​TCA​ATT​GGT​TGC​TCT​GTG​A
covid_31	GGT​GTT​GTT​TGT​ACA​GAA​ATT​GAC​CC	AGC​CAC​CAC​ATC​ACC​ATT​TAA​GT
covid_32	AAT​TTG​CTG​ATG​ATT​TAA​ACC​AGT​TAA​CTG	CGC​GTC​CTC​TGA​CTT​CAG​TAC​A
covid_33	GGT​GTA​TAC​GTT​GTC​TTT​GGA​GCA	TGT​GTG​GCC​AAC​CTC​TTC​TGT​A
covid_34	ACT​ACC​GAA​GTT​GTA​GGA​GAC​ATT​ATA​CT	ACA​CCG​TGT​AAC​TAT​GTT​AGT​AGT​TGT
covid_35	AGT​GTC​CCT​TGG​GAT​ACT​ATA​GCT	AAA​TGA​AGC​CTC​TAG​ACA​AAA​TTT​ACC​G
covid_36	AAT​TCT​AGA​ATT​AAA​GCA​TCT​ATG​CCG​AC	ACC​AGT​ACA​GTA​GGT​TGC​AAT​AGT​G
covid_37	GGC​ATG​CCT​TCT​TAC​TGT​ACT​GG	TGC​AGC​CAA​TCC​AAG​TAC​ATA​GAA
covid_38	TTT​GGC​TTA​GTT​GCA​GAG​TGG​T	ACA​ACC​GTC​TAC​AAC​ATG​CAC​A
covid_39	CCC​GAT​TTC​AGC​TAT​GGT​TAG​AAT​GT	ACT​GTA​GTG​ACA​AGT​CTC​TCG​CA
covid_40	TTG​TGT​TAA​TTG​TGA​TAC​ATT​CTG​TGC​T	AGG​CAA​TGA​ACC​TTT​AGT​GTT​ATT​AGC​T
covid_41	AGC​TGG​TCA​AAA​GAC​TTA​TGA​AAG​ACA	AAC​ATT​TTA​ACT​GCA​ACT​TCC​GCA​C
covid_42	ACA​GTC​AGC​TTA​TGT​GTC​AAC​CTA​T	CTG​AAT​CAA​CAA​ACC​CTT​GCC​G
covid_43	GCA​GAA​GCT​GAA​CTT​GCA​AAG​AA	TGA​CTT​TTT​GCT​ACC​TGC​GCA​T
covid_44	TGA​AAA​CAT​GAC​ACC​CCG​TGA​C	TTA​CCA​CCC​TTA​AGT​GCT​ATC​TTT​GT
covid_45	GAA​TAA​CTT​ACC​TTT​TAA​GTT​GAC​ATG​TGC	TCA​CGA​GTG​ACA​CCA​CCA​TCA​A
covid_46	ACA​CCT​GTT​CAT​GTC​ATG​TCT​AAA​CA	TTG​TGC​GTA​ATA​TCG​TGC​CAG​G
covid_47	GCC​CAT​TGA​TTG​CTG​CAG​TCA​T	CGT​GTG​TCA​GGG​CGT​AAA​CTT​T
covid_48	TGG​TAA​GCC​AGT​ACC​ATA​TTG​TTA​TGA	TCT​ACA​CCA​CAG​AAA​ACT​CCT​GGT
covid_49	GCT​GGT​GTT​TGT​GTA​TCT​ACT​AGT​GG	GGC​AAC​TAC​ATG​ACT​GTA​TTC​ACC​A
covid_50	TCG​TAG​TAA​CAT​GCC​TTG​CCT​AC	CCA​GAA​AGG​TAC​TAA​AGG​TGT​GAA​CA
covid_51	CCA​GTT​TAC​TCA​TTC​TTA​CCT​GGT​GT	TTA​ACA​AAA​AGG​TGC​ACA​GCG​C
covid_52	TGG​TTC​TTT​AGT​AAT​TAC​CTA​AAG​AGA​CGT	TGA​GAG​CCT​TTG​CGA​GAT​GAC​A
covid_53	AGT​ACA​AGT​ATT​TTA​GTG​GAG​CAA​TGG​A	AGC​ATG​TCT​TCA​GAG​GTG​CAG​A
covid_54	ACA​CTT​AAC​GGT​CTT​TGG​CTT​GA	CTG​TCC​TGG​TTG​AAT​GCG​AAC​A
covid_55	TGT​ACT​TAA​GCT​TAA​GGT​TGA​TAC​AGC​C	ACC​TTC​TAA​GTC​TGT​GCC​AGC​A
covid_56	ACT​GTG​TCT​CTT​TTT​GTT​ACA​TGC​AC	GTC​AAC​ATG​GTC​TTG​TGT​TAG​AGG​T
covid_57	GTT​TCT​CAA​TCG​ATT​TAC​CAC​AAC​TCT	AAC​CAG​TGG​TGT​GTA​CCC​TTG​A
covid_58	TGT​TGT​TAG​ACA​ATG​CTC​AGG​TGT	AGC​TAC​AGT​GGC​AAG​AGA​AGG​T
covid_59	TGC​TAT​GGG​TAT​TAT​TGC​TAT​GTC​TGC	GTC​CAC​ACT​CTC​CTA​GCA​CCA​T
covid_60	ACT​GTG​TTA​TGT​ATG​CAT​CAG​CTG​T	AGA​AAA​TAG​GGC​AAT​ACT​CAA​CAC​ACA
covid_61	TCT​CTG​TTA​CTT​CTA​ACT​ACT​CAG​GTG​T	TGC​TAT​TCT​TGG​GTG​GGA​GTA​GT
covid_62	ACT​GAC​TCT​TGG​TGT​TTA​TGA​TTA​CTT​AGT	CTG​GAC​ACA​TTG​AGC​CCA​CAA​T
covid_63	TGC​ACA​TCA​GTA​GTC​TTA​CTC​TCA​GT	AGC​TGC​ATA​TGA​TGG​AAG​GGA​AC
covid_64	AGC​TTT​GTG​AAG​AAA​TGC​TGG​ACA	TTG​CCC​TCT​TGT​CCT​CAG​ATC​T
covid_65	CGT​AAG​TTG​GAA​AAG​ATG​GCT​GAT​C	GGA​TTT​CCC​ACA​ATG​CTG​ATG​C
covid_66	TGG​TTG​TCA​TAC​CAG​ACT​ATA​ACA​CAT	TAG​TAC​CGG​CAG​CAC​AAG​ACA​T
covid_67	GCT​TTA​AGG​GCC​AAT​TCT​GCT​G	CCT​ACA​AGG​TGG​TTC​CAG​TTC​TG
covid_68	GGA​TTT​GAA​ATG​GGC​TAG​ATT​CCC​T	TTG​GTT​GTC​CCC​CAC​TAG​CTA​G
covid_69	GCC​AAT​TCA​ACT​GTA​TTA​TCT​TTC​TGT​GC	CCC​ACA​GGG​TCA​TTA​GCA​CAA​G
covid_70	CTG​TAC​TGC​CGT​TGC​CAC​ATA​G	GTA​AGA​CGG​GCT​GCA​CTT​ACA​C
covid_71	GAA​CCC​ATG​CTT​CAG​TCA​GCT​G	GCA​ACA​GCT​GGA​CAA​TCC​TTA​AGT
covid_72	TAC​TTT​GTA​GTT​AAG​AGA​CAC​ACT​TTC​TCT	GGG​TTT​TCT​ACA​AAA​TCA​TAC​CAG​TCC​T
covid_73	TGT​GAC​ACA​TTA​AAA​GAA​ATA​CTT​GTC​ACA	ACC​TGG​CGT​GGT​TTG​TAT​GAA​A
covid_74	TGG​TAT​TGT​TGG​TGT​ACT​GAC​ATT​AGA	TGG​GTG​GTA​TGT​CTG​ATC​CCA​A
covid_75	TGG​GAT​TTG​TTA​AAA​TAT​GAC​TTC​ACG​G	ACA​CCT​AGC​TCT​CTG​AAG​TGG​T
covid_76	CAA​GTT​TTG​GAC​CAC​TAG​TGA​GAA​AAA	CCG​GGT​TTG​ACA​GTT​TGA​AAA​GC
covid_77	ATG​CAC​GCT​GCT​TCT​GGT​AAT​C	AGT​TGT​CTG​ATA​TCA​CAC​ATT​GTT​GGT
covid_78	TCT​TTG​CTC​AGG​ATG​GTA​ATG​CT	TGC​GAA​AAG​TGC​ATC​TTG​ATC​CT
covid_79	CAA​CAA​CCT​AGA​CAA​ATC​AGC​TGG​T	CTC​CTC​TAG​TGG​CGG​CTA​TTG​A
covid_80	CCG​TAG​CTG​GTG​TCT​CTA​TCT​GT	ACA​CGT​TGT​ATG​TTT​GCG​AGC​A
covid_81	CCT​AAA​TGT​GAT​AGA​GCC​ATG​CCT	TGC​ATT​AAC​ATT​GGC​CGT​GAC​A
covid_82	CAT​CAG​GAG​ATG​CCA​CAA​CTG​C	AGC​CAC​TAG​ACC​TTG​AGA​TGC​A
covid_83	ACA​TTT​CTC​AAT​GAT​GAT​ACT​CTC​TGA​CG	GGC​CCC​TAG​GAT​TCT​TGA​TGG​A
covid_84	ACA​TAC​AAT​GCT​AGT​TAA​ACA​GGG​TGA	ACA​TGT​GTC​CTG​TTA​ACT​CAT​CAT​GT
covid_85	ATG​CTT​ACC​CAC​TTA​CTA​AAC​ATC​CT	GGT​CTA​CGT​ATG​CAA​GCA​CCA​C
covid_86	ACC​GCA​TAC​AGT​CTT​ACA​GGC​T	AGC​ACA​CAA​TGG​AAA​ACT​AAT​GGG​T
covid_87	GAT​GTG​ACT​CAA​CTT​TAC​TTA​GGA​GGT	ACA​GCA​CTT​CAC​GTA​CAG​TAG​C
covid_88	AAG​CTT​TTT​GCA​GCA​GAA​ACG​C	GTT​GTA​CCT​CGG​TAA​ACA​ACA​GCA
covid_89	TGG​TTA​TCG​TGT​AAC​TAA​AAA​CAG​TAA​AGT	GGT​GGT​CCC​TGG​AGT​GTA​GAA​T
covid_90	CTC​AGA​TGA​GTT​TTC​TAG​CAA​TGT​TGC	TCA​AAA​CAC​TCT​ACA​CGA​GCA​CG
covid_91	GAT​GCA​CTA​TGT​GAG​AAG​GCA​TTA​AAA	GTG​CAG​GTA​ATT​GAG​CAG​GGT​C
covid_92	TGA​TTT​GAG​TGT​TGT​CAA​TGC​CAG​A	ACA​TCA​TGC​GTG​ATA​ACA​CCC​T
covid_93	CAC​TGT​GAG​TGC​TTT​GGT​TTA​TGA​T	GAG​CCC​TGT​GAT​GAA​TCA​ACA​GT
covid_94	TGG​AGA​AAA​GCT​GTC​TTT​ATT​TCA​CCT	TGT​AAA​GTT​GCC​ACA​TTC​CTA​CGT
covid_95	TTA​CCA​GAG​CAA​AAG​TAG​GCA​TAC​T	TCA​TGT​CCT​TAG​GTA​TGC​CAG​GT
covid_96	TAC​ACA​GGC​ACC​TAC​ACA​CCT​C	AGG​TAC​AGC​AAC​TAG​GTT​AAC​ACC
covid_97	GAG​GGG​TGT​CAT​GCT​ACT​AGA​GA	TGT​CAA​CTC​AAA​GCC​ATG​TGC​C
covid_98	CCT​TGG​AAT​GTA​GTG​CGT​ATA​AAG​ATT​G	TGG​TTG​CTT​TGT​AGG​TTA​CCT​GT
covid_99	ATT​GGA​TTT​GAT​TAC​GTC​TAT​AAT​CCG​TTT	CCA​ATG​TCG​TGA​AGA​ACT​GGG​A
covid_100	ATG​CGG​CTT​GTA​GAA​AGG​TTC​A	CGA​CAT​TGC​AAT​TCC​AAA​ATA​GGC​A
covid_101	CCT​TGT​AGT​GAC​AAA​GCT​TAT​AAA​ATA​GAA​G	ACA​TGG​ACT​GTC​AGA​GTA​ATA​GAA​AAA​TG
covid_102	CAT​TCC​ACA​CAC​CAG​CTT​TTG​ATA​A	AAA​CCC​ACA​AGC​TAA​AGC​CAG​C
covid_103	AGA​CAT​CAT​GCT​AAT​GAG​TAC​AGA​TTG​T	GCC​CAA​AGC​TCA​AAT​GCT​ACA​TT
covid_104	ACA​CAA​AAG​TTG​ATG​GTG​TTG​ATG​T	CAG​TGA​GTG​GTG​CAC​AAA​TCG​T
covid_105	GCT​CCA​GCA​CAT​ATA​TCT​ACT​ATT​GGT	ACT​GTG​TTT​TTA​CGG​CTT​CTC​CA
covid_106	ACA​ACC​ATC​TGT​AGG​TCC​CAA​AC	TCA​GTA​GAT​GTA​AAC​CAC​CTA​ACT​GAC​T
covid_107	GGT​ATA​AAT​TAG​AAG​GCT​ATG​CCT​TCG	CTT​TGA​CAA​CCT​TAG​AAA​CTA​CAG​ATA​AAT​C
covid_108	CGC​AAA​CAG​GTT​CAT​CTA​AGT​GTG	GCC​TTT​AGG​TAA​TGT​TGC​ACT​ATC​AC
covid_109	TGC​TAT​GCC​TAA​TCT​TTA​CAA​AAT​GCA	GAT​CTG​AAT​CGA​CAA​GCA​GCG​T
covid_110	AAA​GGA​GTT​GCA​CCA​GGT​ACA​G	ATA​GCC​ACG​GAA​CCT​CCA​AGA​G
covid_111	TGT​TAC​AAA​AGA​AAA​TGA​CTC​TAA​AGA​GGG	TAA​CCA​TCT​ATT​TGT​TCG​CGT​GGT
covid_112	AAT​GTG​AAT​GCG​TCA​TCA​TCT​GAA​G	GAA​CAT​CAC​TAG​AAA​TAA​CAA​CTC​TGT​TGT
covid_113	ACT​GCT​GTT​ATG​TCT​TTA​AAA​GAA​GGT​CA	CAG​GGT​AAT​AAA​CAC​CAC​GTG​TGA
covid_114	AGT​CAG​TGT​GTT​AAT​CTT​ACA​ACC​AGA	TGT​TAG​ACT​TCT​CAG​TGG​AAG​CAA​A
covid_115	TGG​GAC​CAA​TGG​TAC​TAA​GAG​GT	ACT​CTG​AAC​TCA​CTT​TCC​ATC​CAA​C
covid_116	CGC​TAC​TAA​TGT​TGT​TAT​TAA​AGT​CTG​TGA	CCT​GAG​GGA​GAT​CAC​GCA​CTA​A
covid_117	AGG​GAA​TTT​GTG​TTT​AAG​AAT​ATT​GAT​GGT	GCA​CAG​TCT​ACA​GCA​TCT​GTA​ATG​G
covid_118	TGT​GGG​TTA​TCT​TCA​ACC​TAG​GAC​T	AAC​AGA​TGC​AAA​TCT​GGT​GGC​G
covid_119	AGA​GTC​CAA​CCA​ACA​GAA​TCT​ATT​GT	CCC​TGG​AGC​GAT​TTG​TCT​GAC​T
covid_120	TGA​TCT​CTG​CTT​TAC​TAA​TGT​CTA​TGC​A	TGT​GCT​ACC​GGC​CTG​ATA​GAT​T
covid_121	AAC​AAT​CTT​GAT​TCT​AAG​GTT​GGT​GGT	TAG​GTC​CAC​AAA​CAG​TTG​CTG​G
covid_122	GGT​TTC​CAA​CCC​ACT​AAT​GGT​GT	TCA​AGT​GTC​TGT​GGA​TCA​CGG​A
covid_123	ACA​AAA​AGT​TTC​TGC​CTT​TCC​AAC​A	CCC​CTA​TTA​AAC​AGC​CTG​CAC​G
covid_124	CAA​CTT​ACT​CCT​ACT​TGG​CGT​GT	AAA​ATT​TGT​GGG​TAT​GGC​AAT​AGA​GTT
covid_125	TGC​CTA​CAC​TAT​GTC​ACT​TGG​TG	CTT​GTG​CAA​AAA​CTT​CTT​GGG​TGT
covid_126	TGG​CAG​TTT​TTG​TAC​ACA​ATT​AAA​CCG	AGC​AGC​AAT​ATC​ACC​AAG​GCA​A
covid_127	ACT​TTT​CAA​CAA​AGT​GAC​ACT​TGC​A	AGA​ACA​TTC​TGT​GTA​ACT​CCA​ATA​CCA
covid_128	CAG​GTG​CTG​CAT​TAC​AAA​TAC​CAT​T	GCC​TCA​ACT​TTG​TCA​AGA​CGT​GA
covid_129	ACG​CTT​GTT​AAA​CAA​CTT​AGC​TCC​A	ATG​AGG​TGC​TGA​CTG​AGG​GAA​G
covid_130	GTC​AGA​GTG​TGT​ACT​TGG​ACA​ATC​A	ACA​ACA​TCA​CAG​TTA​CCA​GAC​ACA
covid_131	TGG​CAC​ACA​CTG​GTT​TGT​AAC​A	AGG​CGG​TCA​ATT​TCT​TTT​TGA​ATG​T
covid_132	TCA​CCA​GAT​GTT​GAT​TTA​GGT​GAC​A	GCA​GCA​GGA​TCC​ACA​AGA​ACA​A
covid_133	TGG​TGA​CAA​TTA​TGC​TTT​GCT​GTA​TG	GCA​ACG​CCA​ACA​ATA​AGC​CAT​C
covid_134	CTC​CTT​CAG​ATT​TTG​TTC​GCG​C	GAT​AGA​GAA​AAG​GGG​CTT​CAA​GGC
covid_135	GGG​TGT​TCA​CTT​TGT​TTG​CAA​CT	GAC​TTG​TTG​TGC​CAT​CAC​CTG​A
covid_136	GCC​AAC​TAT​TTT​CTT​TGC​TGG​CA	ACA​TGT​TCA​ACA​CCA​GTG​TCT​GT
covid_137	TGT​ATT​ACA​CAG​TTA​CTT​CAC​TTC​AGA​CT	CGT​ACC​TGT​CTC​TTC​CGA​AAC​G
covid_138	GAC​GAC​TAC​TAG​CGT​GCC​TTT​G	TCG​TTT​AGA​CCA​GAA​GAT​CAG​GAA​CT
covid_139	ACG​TGA​GTC​TTG​TAA​AAC​CTT​CTT​TTT	TGG​CAT​AGG​CAA​ATT​GTA​GAA​GAC​A
covid_140	AAG​CTC​CTT​GAA​CAA​TGG​AAC​CT	AAT​GAC​CAC​ATG​GAA​CGC​GTA​C
covid_141	TAG​GCT​TGA​TGT​GGC​TCA​GCT​A	AGC​GTT​CGT​GAT​GTA​GCA​ACA​G
covid_142	TTG​CTG​GAC​ACC​ATC​TAG​GAC​G	ACC​TGA​AAG​TCA​ACG​AGA​TGA​AAC​A
covid_143	ACA​CAG​ACC​ATT​CCA​GTA​GCA​GT	AGC​GAG​TGT​TAT​CAG​TGC​CAA​G
covid_144	TGA​AGA​GCA​ACC​AAT​GGA​GAT​TGA	GTG​TTT​TAC​GCC​GTC​AGG​ACA​A
covid_145	TCC​TCT​AGC​TGA​TAA​CAA​ATT​TGC​ACT	AGC​AGA​AAG​GCT​AAA​AAG​CAC​AAA
covid_146	TGC​TTC​ACA​CTC​AAA​AGA​AAG​ACA​GA	AGG​ACA​CGG​GTC​ATC​AAC​TAC​A
covid_147	ACA​ACT​GTA​GCT​GCA​TTT​CAC​CA	ACG​AAC​AAC​GCA​CTA​CAA​GAC​T
covid_148	ACC​CAT​TCA​GTA​CAT​CGA​TAT​CGG​T	ACT​GCC​AGT​TGA​ATC​TGA​GGG​T
covid_149	TAA​TGG​ACC​CCA​AAA​TCA​GCG​A	CGT​CTG​GTA​GCT​CTT​CGG​TAG​T
covid_150	CGA​GGA​CAA​GGC​GTT​CCA​ATT​A	CGA​TTG​CAG​CAT​TGT​TAG​CAG​G
covid_151	TGC​AAC​TGA​GGG​AGC​CTT​GAA​T	TCA​ATC​TGT​CAA​GCA​GCA​GCA​A
covid_152	GGA​ACT​TCT​CCT​GCT​AGA​ATG​GC	GTC​TGA​TTA​GTT​CCT​GGT​CCC​CA
covid_153	TAA​CAC​AAG​CTT​TCG​GCA​GAC​G	TAG​GCT​CTG​TTG​GTG​GGA​ATG​T
covid_154	TCC​AAA​TTT​CAA​AGA​TCA​AGT​CAT​TTT​GC	CCT​TGT​GTG​GTC​TGC​ATG​AGT​T
covid_155	TTC​TCC​AAA​CAA​TTG​CAA​CAA​TCC​A	CGG​TGA​AAA​TGT​GGT​GGC​TCT​T
covid_156	ACT​CTT​GTG​CAG​AAT​GAA​TTC​TCG​T	AAG​CTA​TTA​AAA​TCA​CAT​GGG​GAT​AGC​A

### 2.6 Taqman assay

As there were subgenomic amplicons detected at low viral titres at Day 13 and 15 that reflected non-specific amplification with SyBr Green qPCR using ARTIC primers, we wanted to compare how one-step Taqman qRT-PCR assay fared compared to RT followed by SyBr Green qPCR. Taqman PCR were designed with one primer in the TRS-L and the other primer and the Taqman probe located within the subgenomic fragment about 20,000 nuclceotides away. Taqman probes resulted in better concordance between subgenomic RNA and genomic RNA and can be is empirically more sensitive compared to the two-step qRT-PCR as seen from the ability to detect the subgenomic RNA on P07 Day 12 (Supplementary Figure S3).

Real time probe qPCR measurements were performed Applied Biosystems QuantStudio system. Primers were designed to span 7b-mrna (seq_143) and E-mRNA (seq_133) respectively (Table 2) and the experiment was conducted as per TOYOBO THUNDERBIRD™ Probe qPCR Master Mix instructions. For SARS-COV-2 genomic RNA, 1 µl of heat-inactivated RNA was amplified using a 10 µl Fortitude 2.1 mix (MiRXES) as per manufacturer’s instructions (scaled to 10 µl reaction). For subgenomic RNA, 1 µl of heat-inactivated RNA was mixed with 5 µl qPCR mastermix, 0.25 µl DNA polymerase, 0.25µl RT, 0.25 µl 10 µM probe, 0.5 µl 10 µM primers in a 10 µl reaction. For both reactions, the cycling conditions were as followed, 48°C 15 min, 95°C 2 min, and 45 cycles of 95°C 10s, 59°C 30s.

**TABLE 2:** Primers and Taqman probes for Taqman assay.

Name	Sequence
seq_133_reverse	GCC​ATA​ACA​GCC​AGA​GGA​AA
seq_143_reverse	GCC​GTC​AGG​ACA​AGC​AAA​AG
seq_left	ACC​TTC​CCA​GGT​AAC​AAA​CCA
seq_133_internal_probe	[6FAM]AATTTGCCTATGCCAACAGG[BHQ1]
seq_143_internal_probe	[6FAM]TTGGCACTGATAACACTCGC[BHQ1]

### 2.7 Real-time PCR analysis

Quantification of the relative abundance of sub-genomic transcripts to genomic transcripts were performed by obtaining delta Ct change defined by the difference between the geometric mean of the Taqman assay targeting sub-genomic transcripts described above and the fortitude assay. Wilcoxon ranked sum test with continuity correction is then applied to calculate the significance of the delta CT changes across time.

## 3 Results

### 3.1 Amplicon-based sequencing identifies sub-genomic transcript-derived junction reads

**FIGURE 1:**
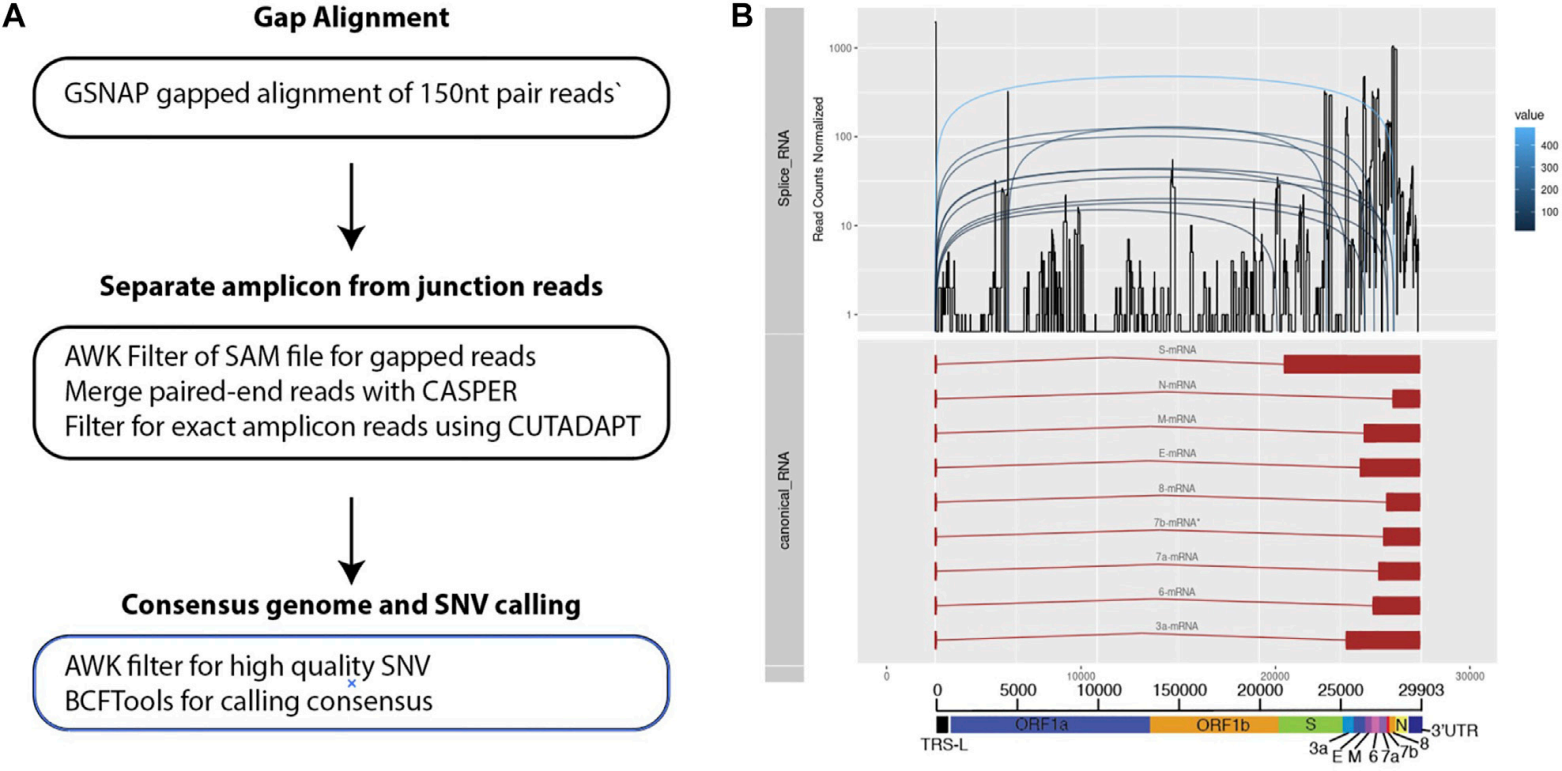
Bioinformatics workflow for identification and visualization of gapped reads and amplicons. **(A)** Three main stages of processing the sequenced data. **(B)** Multiple track visualization of the sequenced data. X-axis corresponds to the COVID genomic length. The top track of the figure reflects the structure of the known and annotated sequences of subgenomic transcripts. The second tracks show an example of the coverage of junctions reads identified using the pipeline specified. Distinct peaks are observed that corresponds to the canonical transcripts on the top track. The third tracks reflect how gapped reads are separated across the subgenomic junctions.

**FIGURE 2:**
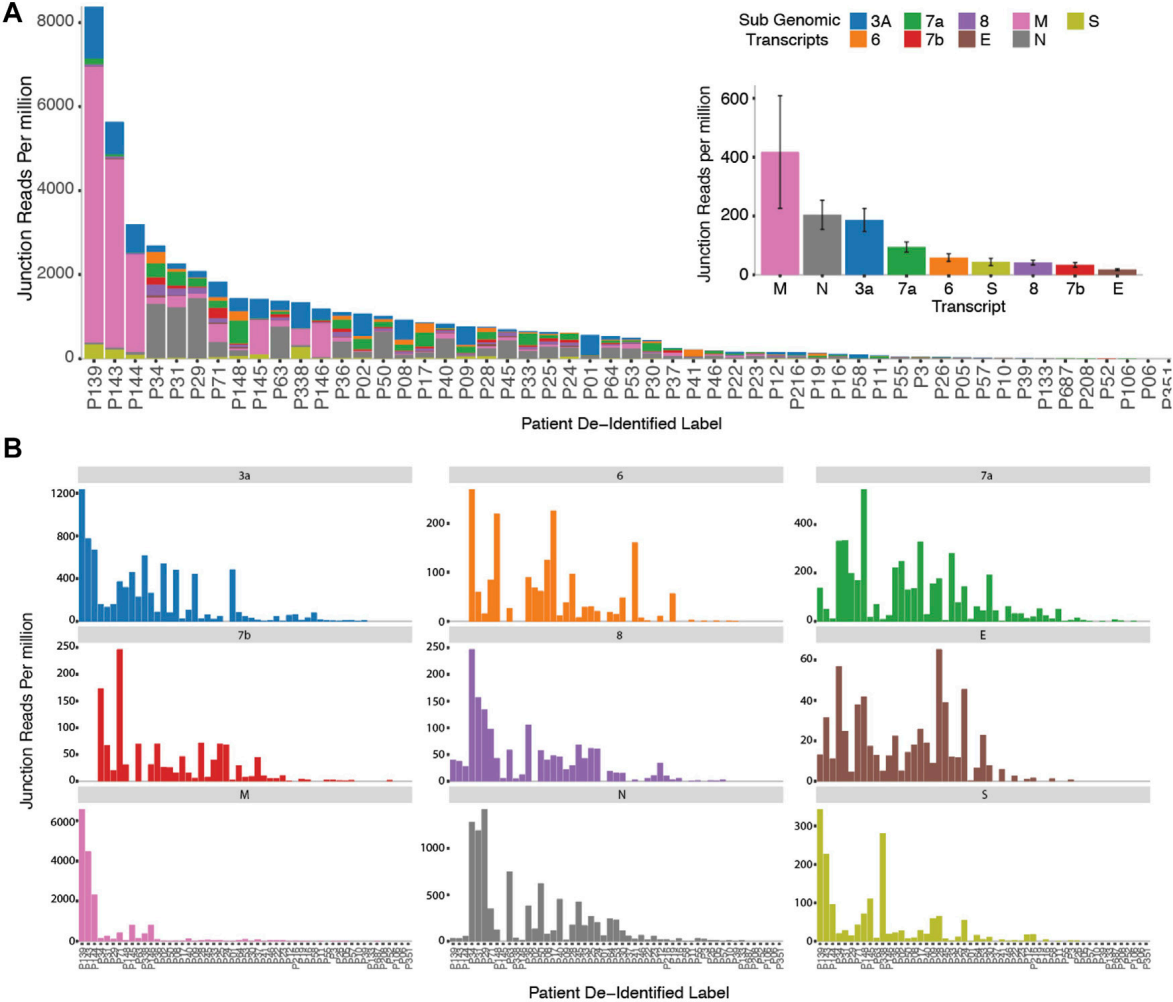
Cohort wide identification of subgenomic Reads. **(A)** Stacked bar graphs reflecting the amount of normalized sub genomic gapped reads for each of the nine canonical subgenomic transcripts across our patient cohort. (Inset) Distribution of total reads per canonical transcript across the patient cohort. **(B)** Distribution of normalized individual subgenomic gapped read counts across the patient cohorts.

In order to probe genomic changes in SARS-CoV-2, we designed 156 primers pairs using the ARTIC protocol (https://artic.network) to generate genome tiling amplicons approximately 300 nt in length to be compatible with 2 × 150bp sequencing chemistry. This protocol allows generation of consensus COVID-19 genome and dentification of single nucleotide variants (Figure 1A). We sequenced samples from 53 SARS-CoV-2 positive patients who were hospitalized at the National University Health System between February to May 2020. We observed that a proportion of sequencing reads does not contain the intended paired primer sequences. These reads span large genomic distances, indicative of gapped reads originating from the splice junction of the subgenomic transcripts (Kim et al., 2020). Alignment of the gap-spanning junction reads to canonical subgenomic transcripts (Figure 1B) identified all nine previously reported canonical subgenomic transcripts (Wölfel et al., 2020), indicating that our amplicon design generated reads composed of leader-body junctions. The genome distribution of junction reads shows a sharp peak at the 5’ end corresponding to the leader sequence and high coverage in the 3’ end for the nested transcript bodies (Figure 1B). These canonical gapped reads made up 0.001%–1% of the total amplicon reads. Estimating the transcript abundance using junction reads across all 53 samples showed varying levels of total subgenomic transcripts across all samples and transcription distribution within each sample (Figures 2A,B). Notably, we see in some samples, a high expression of the envelope encoding gene (M) distinct from that seen in transcripts isolated from cultured virus (Kim et al., 2020).

### 3.2 Tracking abundance of subgenomic transcripts across infection course

**FIGURE 3:**
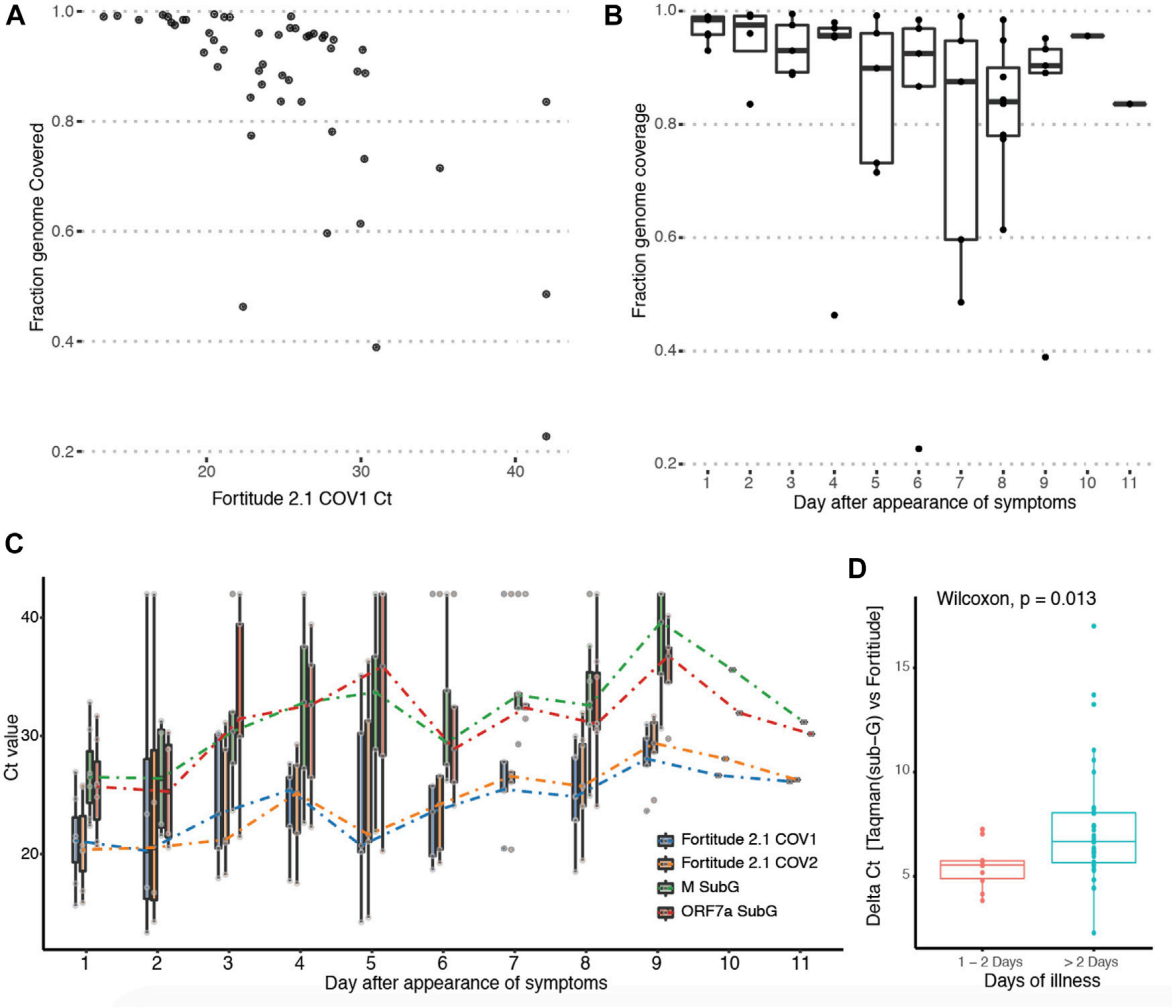
Tracking abundance of subgenomic transcripts across infection course. **(A)** Y-axis: Genome coverage by Arctic sequencing across 53 patients. X-axis: Ct-value of the Taqman assay targeting the genomic region of the SARS-Cov-2 genome from each patient. **(B)** Boxplot of genomic coverage by Arctic sequencing binned by day of symptom at which patient was sampled. **(C)** Boxplot of Taqman quantitation Ct values using primers targeting genomic-specific RNA (Fortitude 2.1 COV1 and COV2) and subgenomic-specific RNA (3a SubG, 7a SubG) binned by day of symptom at which patient was sampled. **(D)** Wilcoxon rank sum test with continuity correction was performed on samples binned by early (day 1 and 2) and latter stages (day 3–11) of infection.

Next, we investigated the effect of viral titre on genome and subgenomic read recovery. As the ORF1ab region targeted by Fortitude 2.1 is not included in any canonical subgenomic RNA, it is actually a good proxy for genomic RNA. We found that the degree of genome read coverage was lower at higher Ct (Figure 3A). This observation is consistent with the hypothesis that high Ct samples are dominated by viral fragments rather than intact viruses (Hu et al., 2020). Comparing genome coverage against the day of illness demonstrated that as the viral load decreased over the course of the infection, the genome coverage generated by our protocol correspondingly decreases (Figure 3B). This suggests an explanation why subgenomic reads were not recovered in samples that were of higher CT values. To explore this more quantitatively, we performed qPCR with Taqman assays (Fortitude 2.1 and custom-designed subgenomic assays targeting ORF5 and ORF7a subgenomic RNA) that specifically target junctional subgenomic transcripts and non-junctional genomic RNA across the 53 patient samples (Figure 3C). We confirmed a general trend where the CT values of both junctional and non-junctional targets increased over the course of the illness. We further observed a small but significant increase in junctional targets CT compared to non-junctional targets in the latter stages of the infection (Figure 3D), suggesting that subgenomic transcripts may possibly serve as an early measure of infection resolution although the variability observed in subgenomic across samples grouped by day of illness was high.

### 3.3 Subgenomic junctional reads captures temporal variation

**FIGURE 4:**
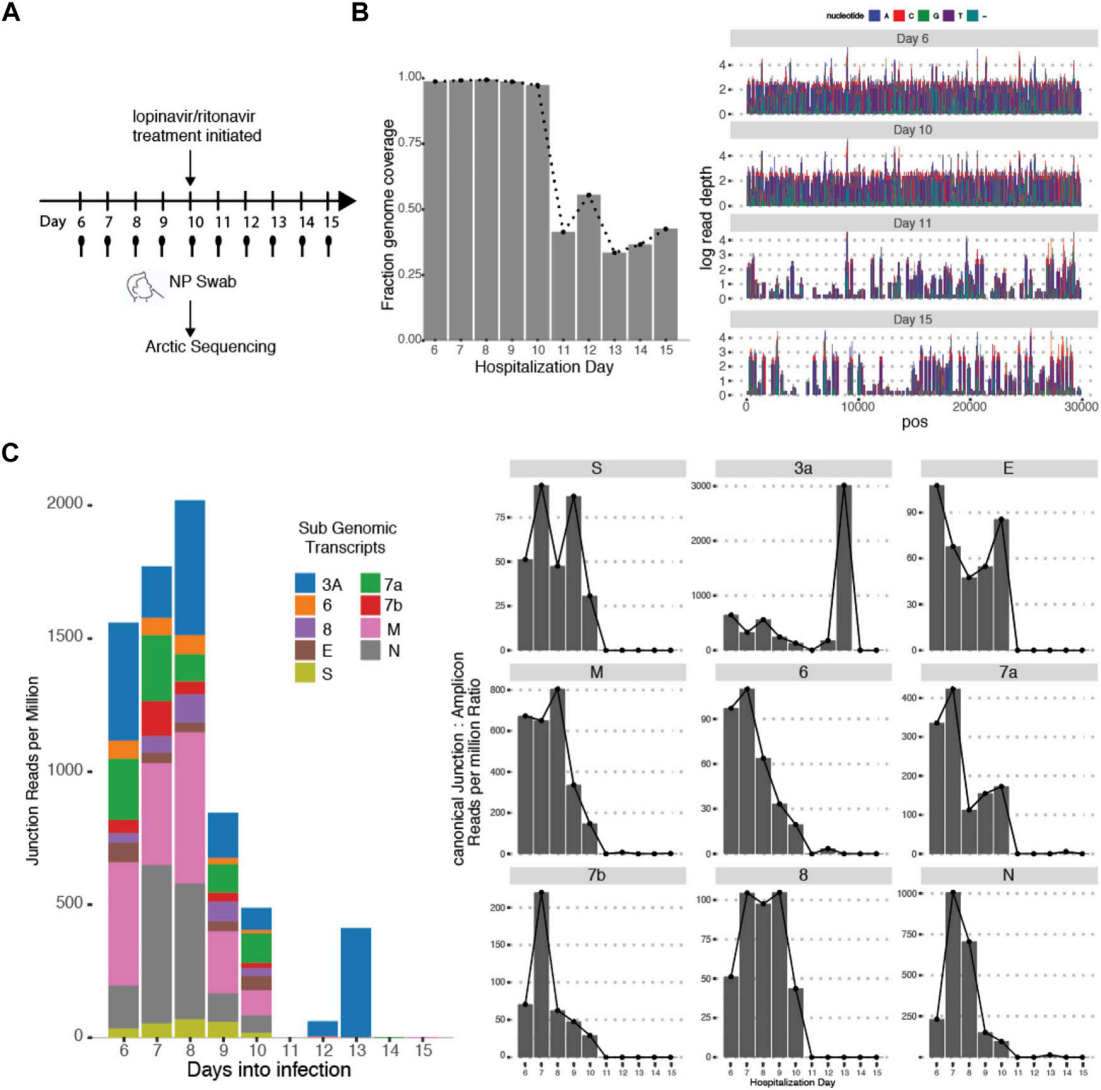
Subgenomic Junctional Reads captures temporal variation. **(A)** Sampling protocol of Patient P07. Patient was sampled daily from day 6 of hospitalization. Patient was treated with Lopinavir/ritonavir from day 10 onwards. **(B)** (left) Genome coverage by Arctic sequencing across the 10 timepoints. (right) Raw read coverage across the genome for Day 6, 10,11,15. Sequencing depth was similar across all timepoints (Supplementary Figure S1) **(C)** (left) Stacked bar graphs reflecting the amount of normalized sub genomic gapped reads for each of the nine canonical subgenomic transcripts across the 10 timepoints. (right) Distribution of normalized individual subgenomic gapped read counts across the 10 timepoints.

**FIGURE 5:**
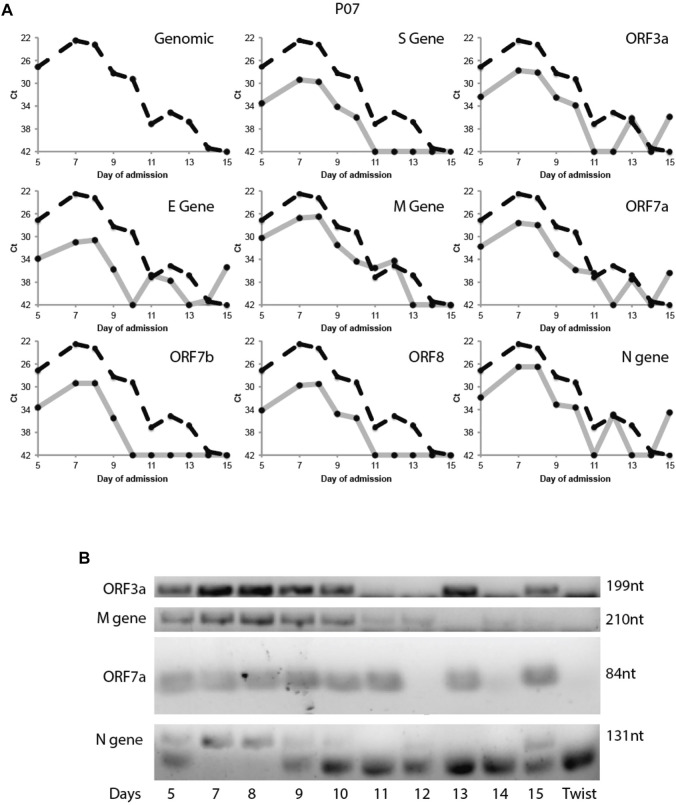
Tracking Patient 07 subgenomic expression from nasopharyngeal swabs across days of admission. Taqman probes were used to amplify a genomic target and eight subgenomic RNA regions specifically target the discontinuous junction *via* real time RT-PCR. **(A)** The Ct values of the subgenomic RNAs (solid grey line) were plotted across 10 time points together with the genomic Ct (dotted black line). **(B)** The PCR product of the above qRT-PCR were verified by gel electrophoresis. Synthetic SARS-CoV-2 genomic RNA (TWIST Bioscience) was used as a negative control.

This variability in subgenomic titres across samples may reflect differences in sample quality, processing or patient-to-patient variation in treatment protocol or intra-host variation or differences in disease trajectory. To better evaluate the utility of subgenomic quantitation in tracking disease progression, we sampled a single patient (P07) daily from day 6 of illness to day 15 (Figure 4A). At each time point, the collected samples were sequenced. Total and subgenomic junctional reads were quantified. The patient was administered lopinavir/ritonavir daily from day 10. We observed that coverage across the genome dropped significantly by more than 50% on day 11 (Figure 4B), the day after antiviral treatment was administered. Notably, subgenomic junction reads across all canonical transcripts also reduced significantly on day 9 and was no longer detectable by day 11 (Figure 4C). Individual subgenomic transcripts showed a similar trend. To verify these, we used amplicon primers that target genomic RNA (dotted line; targeted to N gene region) and junction spanning qPCR (Figure 5A) to quantify genomic and subgenomic RNA targets respectively. For convenience of plotting, undetermined Ct values by qPCR were assigned the value of 42. The amount of subgenomic RNA detected by qPCR tracked the viral genome signal, but fell drastically at day 9 or 10 to undetectable levels on day 11 in agreement with the sequencing data. The amplicon at the completion of the qPCR was then analyzed by agarose gel electrophoresis to verify that amplicons were of the expected sizes (Figure 5B). Importantly, a synthetic SARS-CoV-2 RNA control (Twist) was used as a negative PCR control to demonstrate that the PCR primers were specific for gap-spanning templates as the Twist control template is composed only of synthetic SARS-CoV-2 genome provided as 5 kb fragments. We repeated the analysis with another different longitudinal patient sample (P08) and showed that subgenomic RNA levels tracked genomic RNA similarly, with an earlier decline in the progression of disease (Supplementary Figure S2).

## 4 Discussion

In this article, we present a method for workflow and Taqman probes for the detection of subgenomic RNA which may have diagnostic value in the COVID-19 pandemic, potentially for determining the infectivity of patients. However, further studies will need to be done to validate this hypothesis. Other than ORF1ab, which spans roughly two-thirds of the coronavirus genome and codes for replicase-transcriptase proteins, the other polypeptides including the spike and envelope proteins are dependent on the production of negative strand subgenomic RNA that has the transcription-regulating sequence (TRS) “spliced” next to the translational start site of these polypeptide (Sawicki et al., 2007). This allows transcription of 50–100-fold excess of viral mRNAs that ultimately produce proteins essential for virion formation. Disrupting the formation of N gene subgenomic RNA and consequently N gene mRNA through mutation of conserved RNA motifs effectively curtailed the ability of another coronavirus—the transmissible gastroenteritis virus to produce successful virions (Mateos-Gomez et al., 2013).

Absence of subgenomic RNA in infected cells will likely mean the infected cells are not capable of producing active virions. If active infectious virions are produced and actively transmitting between cells within the host, we hypothesize there will be subgenomic RNA produced. As production of subgenomic RNA is dependent on the intact replicase/transcriptase complex skipping a large portion of the genome and not produced spontaneously from just reverse transcription of the virus, it is distinct from viral fragment residues present in the virus at late stages of infection (Hu et al., 2020) and may be more valuable in detection of infectious SARS-COV-2 patients. Thus, it may be possible to use subgenomic RNA levels to ascertain the presence of ‘active’ infectious virions in which the virions can infect the naïve host cells at the site of sampling. Indeed, when we correlate our subgenomic RNA levels or proportion with day of symptom, we see a correlation that is consistent with the predicted transmissibility of SARS-CoV-2 (He et al., 2020), suggesting that this hypothesis is worth investigating further. There are a few caveats to this hypothesis. Firstly, subgenomic RNAs can self-amplify without the presence of a full-length genomic template, which means the positive strand can act as a template for the negative strand and *vice versa* (Wu and Brian, 2010). However, this requires the presence of ORF1ab proteins that are required for the generation of the subgenomic RNA in the first place, thus subgenomic RNAs are unlikely to persist long after degradation of the genomic template encoding ORF1ab. Second, a similar study on SARS-CoV-2 has suggested that subgenomic RNA content was poorly correlated to day of illness in two patients and thus not a universally useful indicator of viral replication (Alexandersen et al., 2020). They noted the observation that subgenomics samples were more abundantly detected in poor samples enriched in degraded RNA because of a bias for short reads during PCR amplification. This was based on a limited number of samples (n = 2) collected under different conditions and was not conclusive. We performed a controlled time-course experiment where samples were collected from the same patients over the course of 10 days and both sequencing and Taqman quantitation were performed. In this way, differences in subgenomic RNA levels due to sample handling were minimized. We found that subgenomic RNA tracked the genomic RNA over the course of the infection and showed an earlier drop-off that coincided with antiviral treatment.

Lopinavir has shown *in vitro* activity against SARS-CoV-2 replication (Choy et al., 2020), and there was a noticeable reduction in SARS-CoV-2 genome coverage in our patient (P07) following lopinavir/ritonavir initiation, although the subgenomic RNA levels were already in decline a day prior to that. The decline in the viral genome coverage may be due to the activity of the drug, or it may be due to the natural viral kinetics during the disease course. The combination lopinavir/ritonavir is currently not recommended as a treatment regimen for patients with COVID-19 in view of a lack of significant therapeutic effect (WHO, 2023; COVID-19 Treatment Guidelines, 2021). Moving forward, it would be worth using subgenomic RNA levels as a marker for antiviral efficacy in this and other widely used therapeutics in COVID-19 patients such as remdesivir or dexamethasone.

In summary, we demonstrated the ability to detect subgenomic RNA using the ARTIC protocol with low sequence depth and showed that we can track subgenomic distribution and contribution against genomic signal in multiple patients. We showed that there is correlation between subgenomic percentage, subgenomic Ct, genome coverage and genomic Ct with time after symptom first appears. Importantly, we developed a Taqman assay against ORF5 and ORF7a subgenomic RNA that we believe can be used to more closely monitor if subgenomic RNA is reflective of infectivity and can readily be incorporated into other available SARS-COV-2 diagnostics. We believe that this will make a significant contribution to public health efforts to control the virus as well as therapeutic studies evaluating the effects of antiviral therapies.

## Data Availability

The data represented in the study are deposited in the figshare repository, with the accession number: 22060181 and associated doi link: https://doi.org/10.6084/m9.figshare.22060181.v1.
