# Supplementary material for: Sub genomic analysis of SARS-CoV-2 using short read amplicon-based sequencing
Source: Front Genet. 2023 Feb 24;14:1086865. doi: 10.3389/fgene.2023.1086865 (PMC9998678; doi:10.3389/fgene.2023.1086865)
Supplement: Supplementary file 1 [file Table1.DOCX]

Supplementary Material

# Supplementary Figures and Tables

## Supplementary Figures


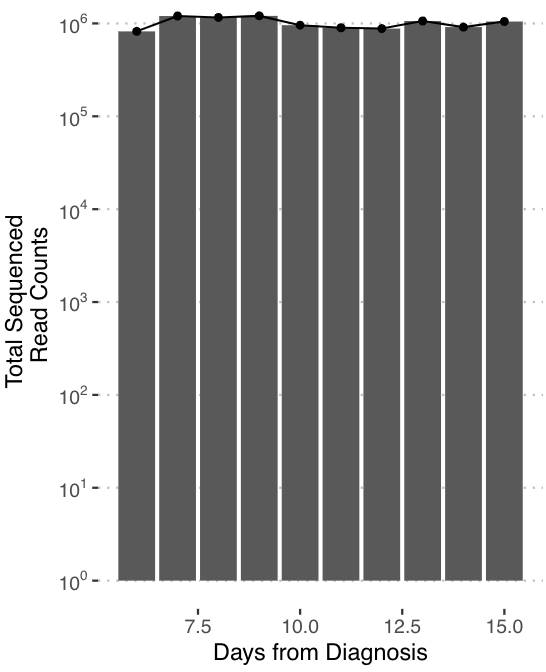


**Supplementary Figure 1.** Sequencing Depth of P07 across different timepoints from Day 6-15.


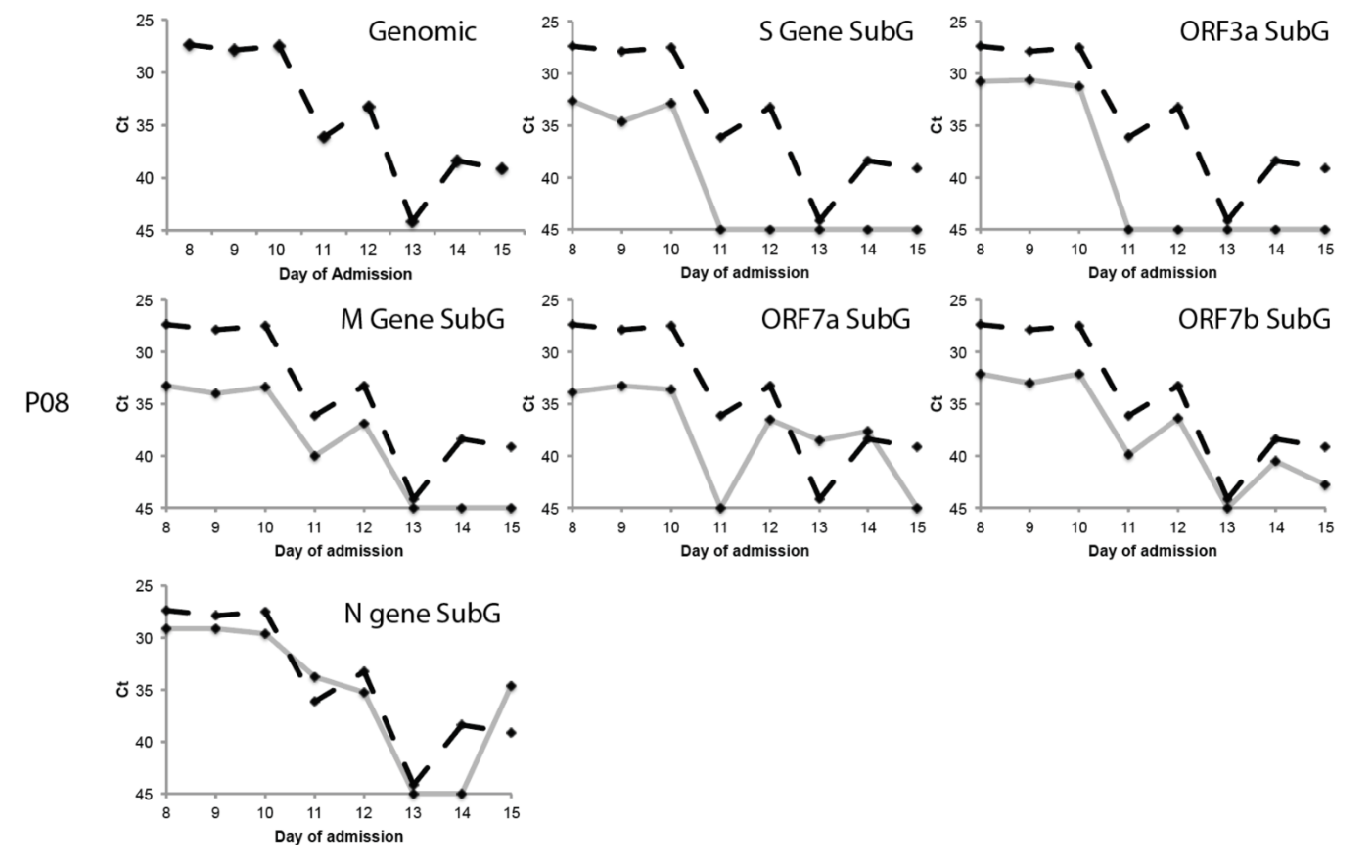

**Supplementary Figure 2.** Tracking Patient 08 subgenomic expression from nasopharyngeal swabs across days of admission. Taqman probes were used to amplify a genomic target and 6 subgenomic RNA regions specifically target the discontinuous junction via real time RT-PCR. The Ct values of the subgenomic RNAs were plotted across 10 time points together with the genomic Ct (dotted line).


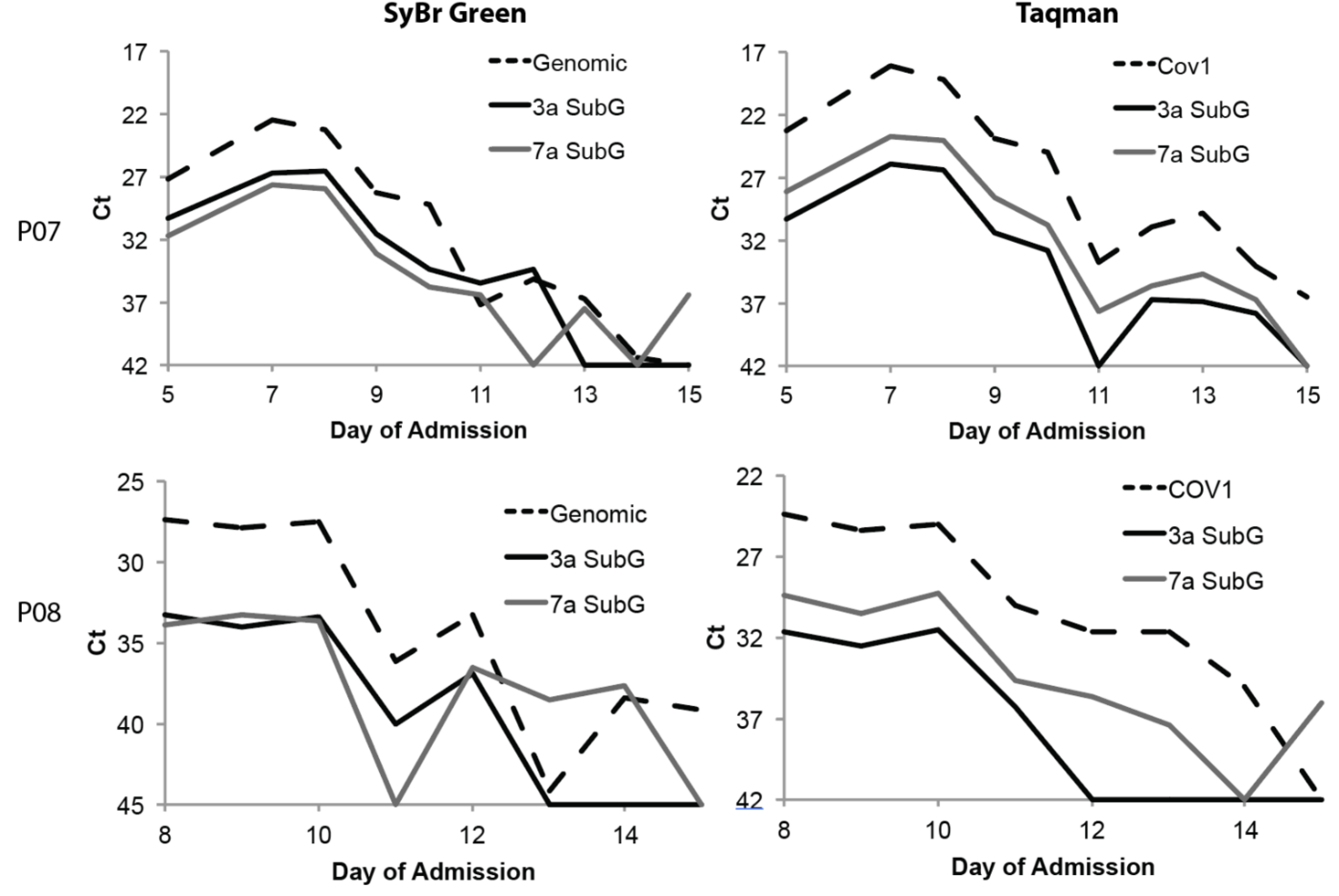


|  | **3a** | | | **7a** | | |
| --- | --- | --- | --- | --- | --- | --- |
|  | Ct | Tm | Detected / Reason | Ct | Tm | Detected / Reason |
| P07 D5 | 30.305 | 80.269 | Yes | 31.708 | 77.831 | Yes |
| P07 D7 | 26.769 | 80.269 | Yes | 27.680 | 77.831 | Yes |
| P07 D8 | 26.533 | 80.117 | Yes | 28.024 | 77.678 | Yes |
| P07 D9 | 31.566 | 80.117 | Yes | 33.192 | 77.678 | Yes |
| P07 D10 | 34.349 | 80.117 | Yes | 35.846 | 77.678 | Yes |
| P07 D11 | 35.506 | 79.964 | Yes | 36.385 | 77.678 | Yes |
| P07 D12 | 34.329 | 80.117 | Yes | Undetermined | 59.999 | No |
| P07 D13 | 37.854 | 75.087 | Wrong Tm | 37.576 | 77.678 | Yes |
| P07 D14 | 35.465 | 77.831 | Wrong Tm | Undetermined | 59.999 | No |
| P07 D15 | 33.923 | 79.964 | Yes | 36.403 | 77.678 | Yes |
| NTC | Undetermined | 75.224 | No | Undetermined | 72.636 | No |
| P08 D8 | 33.284 | 81.361 | Yes | 33.906 | 78.876 | Yes |
| P08 D9 | 33.962 | 81.361 | Yes | 33.308 | 79.337 | Yes |
| P08 D10 | 33.432 | 81.361 | Yes | 33.638 | 78.723 | Yes |
| P08 D11 | 39.977 | 78.287 | Yes | Undetermined | 60.000 | No |
| P08 D12 | 36.869 | 81.207 | Yes | 36.560 | 78.570 | Yes |
| P08 D13 | Undetermined | 75.829 | No | 38.559 | 78.570 | Yes |
| P08 D14 | Undetermined | 76.136 | No | 37.656 | 78.570 | Yes |
| P08 D15 | Undetermined | 75.675 | No | Undetermined | 60.000 | No |
| NTC | Undetermined | 60.000 | No | Undetermined | 60.000 | No |

**Supplementary Figure 3.** Comparison of Ct values between SyBr Green RT-qPCR and one-step Taqman assay in Patient 07 and 08.  The SyBr Green Ct and Melting Temperature (Tm) Is listed below the graphs.


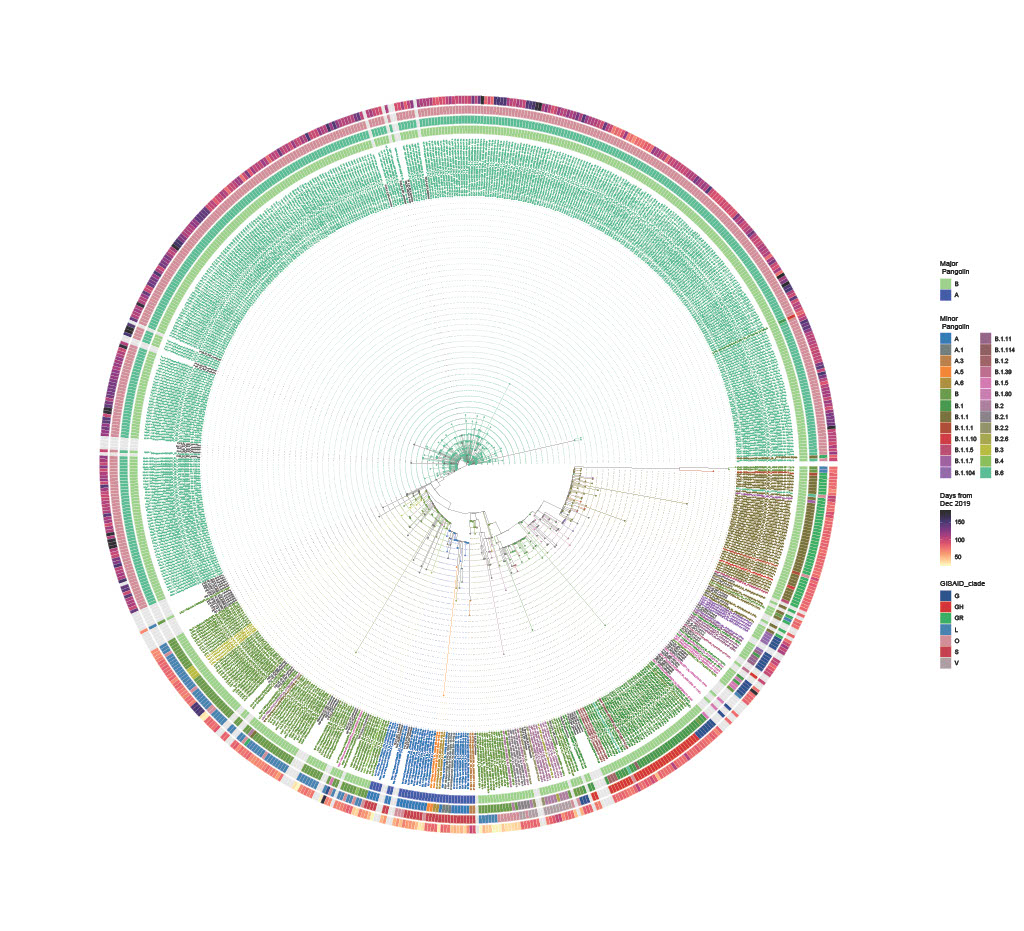


**Supplementary Figure 4.** Tree of samples aligned with other Singapore derived samples available on GISAID at the time of collection. White empty rectangles represents the samples that were collected in our study and the tree depicts where these samples cluster with other samples with known clades.
